# Rhizobacterial Isolates from *Prosopis limensis* Promote the Growth of *Raphanus sativus* L. Under Salt Stress

**DOI:** 10.1007/s00284-023-03379-w

**Published:** 2023-07-05

**Authors:** Rene Flores Clavo, Esteban Valladolid-Suyón, Karin Reinoza-Farroñan, Cristian Asmat Ortega, Pedro Henrique Riboldi Monteiro, Gladys A. Apaza-Castillo, Gabriel Zuñiga-Valdera, Fabiana Fantinatti Garboggini, Sebastian Iglesias-Osores, Carmen Rosa Carreño-Farfán

**Affiliations:** 1 Cesar Vallejo University, Lambayeque, Perú; 2 Department of Biotechnology, Center for Research and Innovation in Multidisciplinary Active Sciences (CIICAM), Pasaje Real Street No 174, Chiclayo, Lambayeque, Perú; 3 Division of Microbial Resources of Chemical, Biological and Agricultural Pluridisciplinary Research Center (CPQBA), University of Campinas (UNICAMP), Alexandre Cazellato No 999, Campinas, Paulínia, São Paulo Brazil; 4 Microbial Biotechnology Research Laboratory, Department of Microbiology and Parasitology, Pedro Ruiz Gallo National University, Juan XXIII No 391 Street, Chiclayo, Lambayeque, Peru; 5 Department of Genetics, “Luiz de Queiroz” College of Agriculture, University of São Paulo (USP), Piracicaba, São Paulo Brazil

## Abstract

Microbial biotechnology employes techniques that rely based on the natural interactions that occur in ecosystems. Bacteria, including rhizobacteria, play an important role in plant growth, providing agricultural crops with an alternative that can mitigate the negative effects of abiotic stress, such as those caused by saline environments. In this study, bacterial isolates were obtained from soil and roots of *Prosopis limensis Bentham* from the department of Lambayeque, Peru. This region has high salinity levels, therefore, the collected samples were used to isolate plant growth-promoting rhizobacteria (PGPR), which were identified through morphological, and physical-biochemical characteristics. These salt tolerant bacteria were screened phosphate solubilization, indole acetic acid, deaminase activity and molecular characterization by 16S rDNA sequencing. Eighteen samples from saline soils of the *Prosopis limensis* plants in the northern coastal desert of San Jose district, Lambayeque, Peru. The bacterial isolates were screened for salt tolerance ranging from 2 to 10%, a total of 78 isolates were found. Isolates 03, 13 and 31 showed maximum salt tolerance at 10%, in vitro ACC production, phosphate solubilization and IAA production. The three isolates were identified by sequencing the amplified 16S rRNA gene and were found to be *Pseudomonas* sp. 03 (MW604823), *Pseudomonas* sp. 13 (MW604824) and *Bordetella* sp. 31 (MW604826). These microorganisms promoted the germination of radish plants and increased the germination rates for treatments T2, T3 and T4 by 129, 124 and 118% respectively. The beneficial effects of salt tolerant PGPR isolates isolated from saline environments can be new species, used to overcome the detrimental effects of salt stress on plants. The biochemical response and inoculation of the three isolates prove the potential of using these strains as a source of products that can be employed for the development of new compounds proving their potential as biofertilizers for saline environments.

**Supplementary Information:**

The online version contains supplementary material available at 10.1007/s00284-023-03379-w.

## Introduction

The salinity in desert soils is a problem reported since pre-Columbian times, representing a limiting factor for agricultural activities in the region of the northern coast of Peru [1]. Approximately 23% of cultivated land in the world is affected by salinity, and 37% is considered sodic soils, considered as anunproductive agricultural land [2], which affects the global productive area [3].

Research points out that the productive bioclimatic zones will change [4], as the abiotic factors will cause a process of desertification and increased salinity rates due to variations in precipitation, dry periods, temperatures, evaporation and light intensity [2]. This is the result of climate change, which is a reality, proved by evidence of the rise of global temperature, and by altered water and rainfall regimes that have been observed and evaluated in recent decades around the world. The climate change threatens our ability to ensure global food security, according to the Food and Agriculture Organization of the United Nations (2023).

This fact, correlated with the physicochemical fluxes of the soil, triggers stress on plants, as high concentrations of salt in the soil decrese the total water potential, caused by ionic and osmotic stress, in which plants induce photosynthesis suppression, delaying the development of the plant, as well as the proliferation of new tissues [5, 6].

Root systems have been reported as key sites of salinity tolerance due to their potential to enhance water and nutrient uptake, as well as limit salt absorption [7].

Plant growth-promoting rhizobacteria (PGPR) can potentially be utilized as a promising alternative and environmentally friendly approach to promote growth and tolerance to salinity stress in plants [8]. The interaction between plants and salt-tolerant PGPR modulates the expression of genes to alleviate the adverse effects of soil salinity on nonsalt-tolerant plants, inducing physiological changes as defence mechanisms [9]. One of these mechanisms involves increased activity of 1-aminocyclopropane-1-carboxylic acid (ACC) deaminase, an enzyme related to the reduction of ethylene concentration and the availability of ammonium in the rhizosphere [10]. Another mechanism is indole-3-acetic acid (IAA) production, which acts upon growth and many other plant physiological responses [11]. Moreover, salinity may lead to nutrient imbalance due to the competition between Na+ and nutrients such as K+ , Ca2+ and Mg2+ or Cl− and NO3- [12]. Usually, salinity-induced phosphorus (P) deficiency in crops is compensated by the addition of P fertilizers; however, 75–90% of P is naturally fixed in the soil [13].

The application of P-solubilizing rhizobacteria contribute to plant growth by increasing the availability of soluble phosphate. There are also other mechanisms that benefits from this strategy: when employing microorganisms to induce plant tolerance to salinity, there is also an alteration of hormones such as jasmonic acid (JA), gibberellic acid (GA), abscisic acid (ABA) and cytokinins (CKs), which also influence plants tolerance to saline environments [14, 15].

Most of these microorganisms are native and help the development of their host plants under stress conditions. The genus *Prosopis* is widely distributed on the Peruvian coast. *Prosopis pallida* (carob), a leguminous tree adapted to arid areas, is an autochthonous species of the dry forests of the northern coasts of Peru and is widely distributed in saline areas. Due to its relevance in the rural economy, most of the previous studies on carob have focused on food applications [16].

Several studies reported that PGPR isolated from *Prosopis laevigata*, such as *Alcaligenes*, *Bacillus*, *Curtobacterium* and *Microbacterium*, significantly improved seed germination and root growth [17]. IAA biosynthesis of *Pseudomonas* strains was further stimulated under salinity conditions, considerably alleviating salt-induced dormancy of wheat seeds [18]. Nitrogen-fixing bacteria are also found in root nodules on *Prosopis alba* [19]. *Arthrobacter koreensis*, identified through 16S rDNA analysis, was found in *Prosopis strombulifera* and is strongly promissory in the synthesis of abscisic acid (ABA), auxins (IAA), gibberellins (GA1, GA3), and jasmonic acid (JA) under adverse environmental conditions such as salt stress [20]. The plant model Radish (*Raphanus sativus*) is an essential root vegetable of the *Brassicaceae* family grown worldwide, and its consumption is due to its nutritional value and because it is easy and fast-growing, it has been applied in microbial biotechnology as a natural, sustainable and economic productive strategy and *Brassica* is widely utilized as an oil and vegetable crop and is harshly affected by abiotic stresses. Therefore, the use of PGPRs along with proper mineral nutrients management can be a strategy to manage the abiotic stresses by improving the biochemical, physiological and growth attributes [21]. And in the case of rhizobacteria, its use in the form of bioproducts has already been shown to be effective [22]. Studies have employed this plant as a model to examine the influence of various priming treatments in order to evaluate the physiological performance (germination, growth, lipid peroxidation, primary and secondary metabolism) and antioxidant activity of radish seedlings, due to its ability to easily accumulate large amounts of nitrate from the soil [23]. Thus, this study aimed to identify PGPR from *Prosopis limensis* by molecular methods and evaluate their effect on the growth of *R. sativus* under salt stress.

## Materials and Methods

### Collection of Soil Samples and Bacterial Isolation

The bacterial strains were retrieved from rhizospheric soil samples of *Prosopis limensis* “carob” trees, located in a forest of San Jose, Lambayeque, Peru (06°45′35.65″ S, 79°57′41.35″ W). *P. limensis* trees of similar phenotypes (height of 5.25–6.10 m) were selected in an ≈ 700 m^2^ forest,.From each tree, three samples of rhizospheric soil were collected. For sampling, a circle was delimited at 0.45 m from the base of the stem, in which three central points were marked. Then, the soil was removed to a depth of approximately 0.60 m, which was enough to reach the lateral roots, then, 100 g of sample was obtained. Samples were transported under refrigeration and sequentially stored at 10 ± 1 °C in the Biotechnology of Center for Research and Innovation in Multidisciplinary Active Sciences (CIICAM Research Center), Peru. Bacteria with phenotypic differences were isolated by serial dilution and cultured in nutrient agar plates with 5% NaCl at 30 °C for up to two days. Then, the isolates were preserved in the collection of extreme microorganisms at the CIICAM Research Center.

### Molecular Identification of the Selected Bacterial Isolates by 16S rRNA Analysis

An isolated colony was used for genomic DNA extraction, following the method described by Pospiech et al.[24], with some modifications. The amplification of the 16S rRNA gene was performed through polymerase chain reaction (PCR). The products were analyzed and visualized by agarose gel electrophoresis and purified with mini columns (GFX PCR DNA & gel band purification kit, GE Healthcare) in an ABI3500XL Series automatic sequencer (Applied Biosystems) according to the manufacturer’s specifications. The detailed protocol conditions used in this section can be found in Flores-Clavo [25].

Partial sequences of the 16S rRNA gene obtained from each isolate were assembled into a contig and then compared with the sequences of organisms represented in the EZBioCloud 16S Database (https://www.ezbiocloud.net) using the “Identify” service [26]. Species assignments were based on closest hits [27]. 16S rRNA gene sequences retrieved from the database and related to the unknown organism gene were selected for alignment in the Clustal X program [28]. Phylogenetic analyses were performed using the Mega version 11.0 program [29], and the phylogenetic tree was constructed from the evolutionary distances calculated by the neighbour-joining method, with bootstrap values from 1000 resamples.

### PGP Trait Characterization and Salinity Tolerance

#### IAA Production

9 × 10^8^ cfu mL^−1^ of bacterial cells were obtained from cultures in 5 mL Dworking & Foster (DF) minimum medium with 0.85 M NaCl at 30 °C for 24 h. Bacterial cultures (5%; 0.25 mL) of each isolate were prepared in trypticase soy broth (TSB) supplemented with 0.01 gL^−1^ L-tryptophan 5 mM. After incubation in the dark at 30 °C and shaking at 150 rpm for 72 h, each culture was centrifuged at 3000 rpm for 5 min and evaluated by colorimetric assay [30]. Then, 0.4 mL of each supernatant was mixed with 1.6 mL of Salkowski reagent for 30 min in the dark. The reaction was considered positive for indole-3-acetic acid (IAA) production when the solution turned pink. IAA concentrations were determined through spectrophotometry at 530 nm and using a standard curve obtained from serial dilutions of 100 ppm of IAA [31].

#### Phosphate Solubilization

The phosphate solubilization capacity was determined through the molybdenum blue colorimetric method [32]. Each isolate (5%; 0.25 mL) were inoculated in 5 mL of National Botanical Research Institute’s Phosphate Broth (NBRIP) with 0.85 M NaCl and incubated at 30 °C under 150 rpm for five days. The cultures was centrifuged at 3000 rpm for 5 min; then, and an aliquot of the supernatant was evaluated by employing the Barton’s reagent. The reaction was considered positive for phosphate solubilization capacity when a blue phosphomolybdate complex was formed. P concentrations were measured through spectrophotometry at 690 nm using a standard curve obtained from serial dilutions of 10 ppm of P.

Additionally, we determined the phosphate solubilization index (PSI) in NBRIP solid medium. Isolates were inoculated in trypticase soy agar (TSA) for 24 h. Then, 10 µL of each broth was inoculated into NBRIP medium supplemented with 1 g L^−1^ tricalcium phosphate and 0.85 M NaCl. The plates were incubated at 30 °C for 96 h, and then, PSI was calculated as the ratio between the halo diameter and the colony diameter (cm) [33].

#### ACC Deaminase Activity

ACC deaminase activity was quantified following the method used [34]. The quantity of α-ketobutyrate produced by hydrolysis of ACC was used to estimate ACC deaminase. A standard curve of α-ketobutyrate ranging between 0.1 and 1.0 nmol at 540 nm and compared with the absorbance of the stock solution of 100 mmol L^−1^ α-ketobutyrate (Sigma–Aldrich Co.) was prepared in 0.1 M Tris–HCl (pH 8.5) and stored at 4 °C.

#### Salinity Tolerance

To evaluate tolerance to salt stress, the strains were tested by growing on modified mineral-based nutrient agar plates (peptone 1 g, K_2_PO_4_ 0.2 g, MgSO_4_, 7H_2_O 0.2 g, Ca SO_4_ 0.1 g, agar 15 g, 1 L distilled water) supplemented with increasing concentrations of NaCl (0–30%, w/v, at intervals of 1%) and incubated at 28 °C. NaCl tolerance was qualitatively verified by turbidity of growth of the isolates [35]. The trials were conducted on soils without and with salinity, each with four treatments, corresponding to a control without bacterial inoculation (T1) and two cultures of three strains (T2 to T4). Three replicates were carried out in each treatment, with a total of 12 experimental units per trial.

#### Effects of PGPR Bacterial Inoculation on *R. sativus*

For the germination of *Raphanus sativus* L. var. Champion, the seeds were inoculated with the three new bacterial species obtained in the present study -two strains belonging to the genus *Pseudomonas* spp. and one *Bordetella* spp. which were applied in the germination trials. First, strains with ACC deaminase activity were selected in order to study their effects on seed germination under salt stress. Seeds were surface sterilized with 75% ethanol for 30 s, then 0.1% HgCl for 7 min, and finally washed with distilled water. Then, 40 seeds were placed on Petri plates containing Dworking & Foster (DF) with 0.85 M NaCl for 1 min; all plates were set with filtered paper containing a solution of distilled water (control) and a solution of 80 mM NaCl (CE = 6.94 dSm^−1^). The seeds were previously sterilized with 3% sodium hypochlorite for 1 min and washed five times with sterilized distilled water [36].

Then, the PGPR strain suspensions in sterile distilled water (1.0–2.0 × 10^8^ cfu mL^−1^) were used for seed inoculation; control seeds were treated with sterile distilled water only. Seeds were soaked at room temperature for 10 h in bacterial suspensions (1 mL) and placed into MS medium (with 0, 100, 200, 250, 300, and 500 mM NaCl). Each plate contained 50 seeds, and each treatment was performed in triplicate [35]. Another control group, in which seeds were not inoculated with the PGPR isolates, was considered for germination assays. Finally, the plates were covered in aluminium foil and kept at 30 °C, and the germination of the plants was assessed 12 days after inoculation.

Two independent trials were used to evaluate the effect of inoculants on radish emergence and development. The experiment contained four treatments: control without microorganisms (T1) and three treatments with microbial inoculants (*Pseudomonas* spp.—T2 and T3 and *Bordetella* spp.—T4) with and without salt stress. All treatments were replicated in triplicate.

The soil used for the greenhouse trials consisted of: 36 kg of non-saline soil (EC = 2.42 dSm-1) collected from an agricultural field in the Lamadrid sector and 36 kg of saline soil (EC = 6.89 dSm-1) collected from an agricultural rice field located on the Panamericana Norte road to Morrope. The salt concentration (EC) was determined in the Analytical Chemistry laboratory of the Faculty of Chemical Engineering and Food Industries. Soils were mixed with compost (4:1) and distributed into 4 kg clay pots at a rate of 3 kg per pot. At the base of each pot, 0.5 kg of gravel was placed internally to facilitate drainage.

The inocula was obtained from the three cultures of interest that were previously induced to produce ACC deaminase [10]. The bacteria were grown in trypticase soy broth with 0.85 M NaCl (~ 5%) at 30 °C with daily manual shaking at 6, 12, 18, and 24 h. The bacterial cultures were centrifuged (3000 rpm) for 5 min, the supernatant was discarded, and the biomass was grown in 5 mL of minimal DF medium with 0.85 M NaCl (~ 5%) and 3 mM ACC and incubated at 30 °C for 24 h. The biomass was concentrated by centrifugation and washed with 0.03 M MgSO_4_ solution, resuspended in the same solution and the concentration was standardized to 1.5 × 10^8^ cel mL^−1^ by turbidimetry with Mc Farland nephelometer [10].

Radish seeds were inoculated by immersion for 3 h in 7.5 mL of the corresponding bacterial inoculum. In the control, seeds were immersed in distilled water (7.5 mL). The inoculated seeds (eight per pot) were sown in two furrows (four in each), at a distance of 1 cm between seeds and a depth of 2 cm. The conditioned pots with the experimental soil were kept under greenhouse conditions, with the necessary irrigation with previously dechlorinated water (24 h). The trial was conducted from November 1 to November 30, 2019, recording the maximum (20–25 °C, minimum (15–18 °C) and average (18–22 °C) temperatures, values obtained by the Meteorological Station of the National University Pedro Ruiz Gallo, located on the farm “El Cienago” in Lambayeque.

10 days after planting, the emerged seedlings were counted and every 5 days. 15 days after planting, the height of the plants was measured. 30 days alfter planting, the height, the number of leaves, the number of roots, and the weight of aerial and root biomass were determined, and the indices of effectiveness (IE) were calculated in percentage [37]:

$${\text{IEI }}\left( \% \right){\mkern 1mu} = {\mkern 1mu} \frac{{{\text{Treatment with inoculation}} - {\text{Control without inoculation }} \times 100{\mkern 1mu} }}{{{\text{Control without inoculation}}}}$$

Analysis of ANOVA, Shapiro–Wilk normality test and Tukey post hoc tests at a 5% confidence level (*p* < 0.05) were performed on germination rates and biometric evaluations of the plant using R software [38].

## Results

### Isolation, Molecular Identification, and Phylogenetic Analysis of Selected Isolates

From a total of 78 obtained pure cultures isolated from *P. limensis* rhizospheric soils (Table S1), we selected three isolates to carry on this study: 03, 13 and 31, which displayed different colony morphologies (Fig. S1). These isolates were selected for molecular identification (Table 1). Through 16S gene analysis, we identified isolates 03 and 13 as *Pseudomonas* spp*.* and isolate 31 as *Bordetella* sp*.*, which were deposited in the GenBank database (GenBank nih.gov) from the National Center for Biotechnology Information (NCBI) and identified with the accession numbers MW604823, MW604824 and MW604826, respectively (Tables S2 e S3).

**Table 1:** Isolation and molecular identification of rhizobacterial isolates

Accesion number	Phenotypic profile											Geographic location	Closest GenBank accession	Similarity (%)	Molecular ID
	Gram staining	Catalase test	Oxidase test	TSI Agar Test	Lysine decarbo-xylation	Citrate	Lactose acidity	Manitol acidity	Ornitine acidity	Sorbitol acidity	Voges–Pros-kauer test				
*Pseudomonas* sp. 03 (MW604823)	−	+	+	K/K	−	+	−	−	−	−	−	6°44′13” S 79°56′35” W	AP013070	98.48	*Pseudomonas* sp*.*
*Pseudomonas* sp. 13 (MW604824)	−	+	+	K/K	−	+	−	−	−	−	−	6°44′05”S 79°56′58”W	BBIS01000088	98.97	*Pseudomonas monteilii*
*Bordetella* sp. 31 (MW604826)	−	+	+	K/K	−	+	−	−	−	−	−	6°44′13”S, 79°56′35”W	LC053647	97.76	*Bordetella muralis*

Closest EZBioCloud (https://www.ezbiocloud.net/) type strains’ accession numbers and their similarity percentages are also shown

**+ - **denotes positivity, − - denotes negative; K - alkaline reaction (red color)

**Fig. 1:**
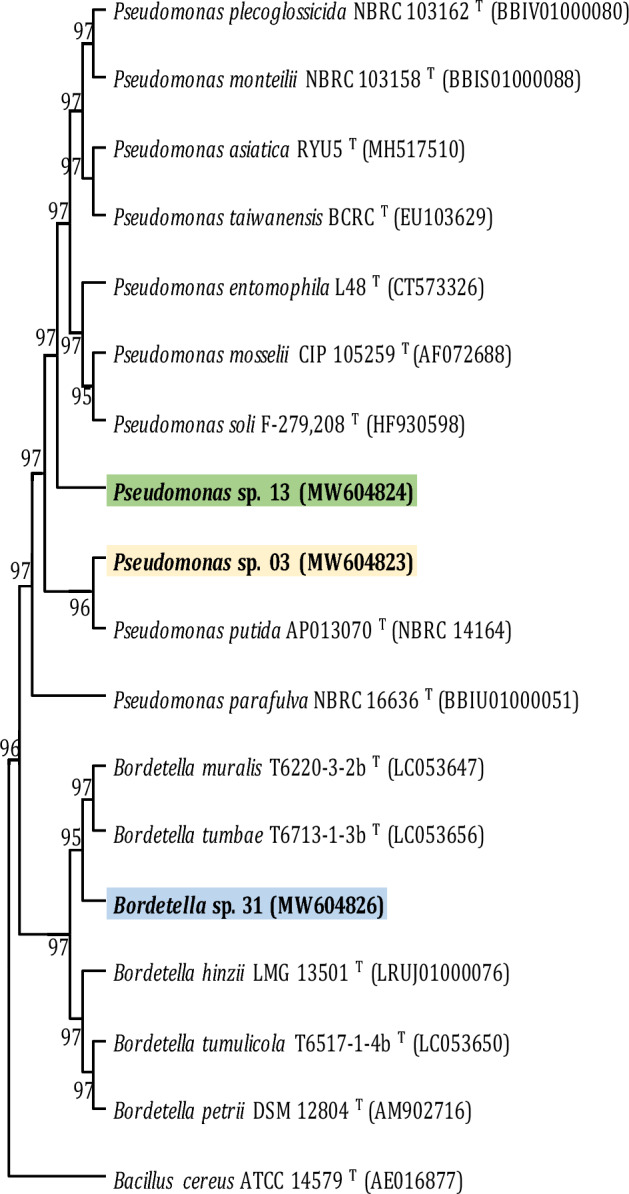
Optimal tree. The evolutionary history was inferred using the neighbor-joining method. The evolutionary distances were computed using the Kimura 2-parameter method and are in units of the number of base substitutions per site. The proportion of sites where at least 1 unambiguous base is present in at least 1 sequence for each descendent clade is shown next to each internal node in the tree. This analysis involved 18 nucleotide sequences with the outgroup *Bacillus cereus* ATCC 14579^ T^ (AE016877). There were a total of 1491 positions in the final dataset. The evolutionary analyses was conducted in MEGA11

The 16S rRNA sequences of the three PGPR strains were analyzed in this study: *Pseudomonas* sp. 03 [Accession number (MW604823)], *Pseudomonas* sp. 13 (MW604824) and *Bordetella* sp. 31 (MW604826) were obtained and compared by MEGA11.0 software. The phylogenetic tree generated from the three strains was mainly divided into three characteristic branches. The samples were constructed against 14 top-hit proximal strain valid names only, according to EZBioCloud, and are presented in Fig. 1.

The sequence of *Pseudomonas* sp. 03 (accession number: MW604823) showed a size of 1466 bp and was blasted with with 100% gene sequence similarity to the type strain *Pseudomonas putida* AP013070^T^ (accession number NBRC 14164), related similarity (98,48%) and the phylogenetic tree bootstrap value 96%. *Pseudomonas* sp. 13 (accession number: MW604824) revealed a size of 1494 bp, that was blasted showing a gene sequence similarity of 93.4% to the type strains *Pseudomonas monteilii* NBRC 103158^T^ (accession number BBIS01000080 with 98,97%), *Pseudomonas plecoglossicida* NBRC 103162^T^ (accession number BBIV01000080) and *Pseudomonas asiatica* RYU5^T^ (accession number MH517510); 98,96% with *Pseudomonas taiwanensis* BCRC 17751^T^ (accession number EU103629) and 98,76% with *Pseudomonas entomophila* L48^T^ (accession number CT573326); the phylogenetic tree bootstrap value was 97%. *Bordetella* sp. 31 (MW604826) sequence showes a size of 1449 bp and was blasted with 98.5% gene sequence identity similarity and were phylogenetically related to the type strain *Bordetella muralis* T6220-3-2b (accession number LC053647); 97,76% similarity and the type strain *Bordetella tumbae* T6713-1-3b (accession number LC053656) (Fig. 1). Most strains were separated by clustering under each subdivided species, generating indications that they may be new species. These phylogenetically characterized lineages have a broad PGPR potential.

### Tolerance to Salt Stress, ACC Deaminase Activity, IAA Production and Phosphate Solubilization

The three possible new species—*Pseudomonas* sp. 03 (MW604823), *Pseudomonas* sp. 13 (MW604824), and *Bordetella* sp. 31 (MW604826), developed on nutrient medium supplemented with 5, 7.5, and 10% NaCl, therefore, the isolates were considered tolerant to salt stress. After all, the isolates showed differential turbidity compared with the control (Figure S2). All isolates showed ACC deaminase activity (Figure S3), IAA production and phosphate solubilization ability, as shown in Table 2 (Fig. S4 and S5).

**Table 2:** Tolerance to salt stress, ACC deaminase activity, indole acetic acid (IAA) and phosphate solubilization of the rhizobacterial isolates

Isolate	Tolerance to NaCl (%)	ACC deaminase activity	IAA production (ppm)	Phosphate solubilization capacity	
				Solubilized phosphorus (ppm)	PSI
*Pseudomonas* sp. 03 (MW604823)	+	+	72.91	28.11	3.6
*Pseudomonas* sp. 13 (MW604824)	+	+	78.02	27.8	3.4
*Bordetella* sp. 31 (MW604826)	+	+	68.91	28.99	3.9

** + **denotes positivity for salt stress tolerance and ACC deaminase activity

### Isolate´s Effect on* R. sativus* Germination

**Fig. 2:**
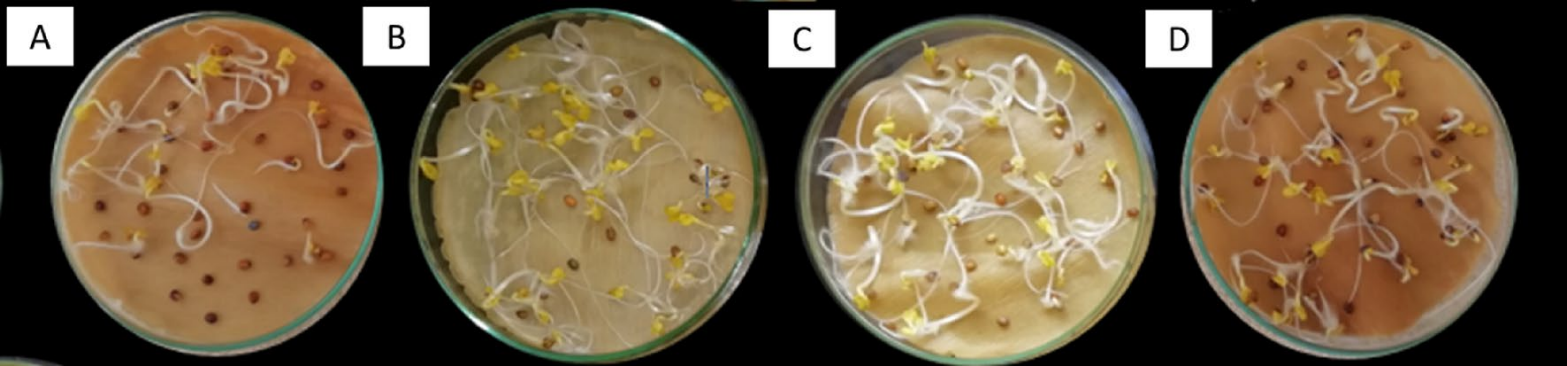
Germinated seeds of inoculated *R. sativus*. From left to right:** A**. Control.** B**. Inoculated with *Pseudomonas* sp. 03 (MW604823);** C**. Inoculated with *Pseudomonas* sp. 13 (MW604824);** D**. Inoculated with *Bordetella* sp. 31 (MW604826)

The percentage of germination after 12 days of inoculation (Fig. 2) ranged 36% for the control, 84.17%, 82.50% and 80.00%, for the strain *Pseudomonas* sp. 03, *Pseudomonas* sp. 13 and *Bordetella* sp. 31, respectively (Table 3).

**Table 3:** Germination percentage of the inoculated *R*. *sativus* seeds

Isolate	Germination (%)
Control	36.67 (± 5.77) b
*Pseudomonas* sp. 03 (MW604823)	84.17 (± 5.20) a
*Pseudomonas* sp. 13 MW604824	82.50 (± 4.33) a
*Bordetella* sp. 31 (MW604826)	80.00 (± 4.33) a

Different letters mean tested treatments and Tukey’s post hoc tests with a confidence level of 5% (*p* < 0.05)

In this study, we were able to prove, by analyzind the difference in germination rate, that the three possible new species can promote the germination of radish plants when compared with the control with increased germination rates for treatments T2, T3 and T4 of 129, 124 and 118% for the strain *Pseudomonas* sp. 03, *Pseudomonas* sp. 13 and *Bordetella* sp. 31, (MW604823, MW604824 and MW604826) respectively.

### Effects of MW604823, MW604824 and MW604826 on the Growth of* R. sativus* Under Salt Stress and Non-Salt Stress

After thirty days of inoculation under salt and non-salt stress conditions, all three strains (MW604823, MW604824 and MW604826) promoted the growth of *R*. *sativus* when comparing to the control (Fig. S6).

**Fig. 3:**
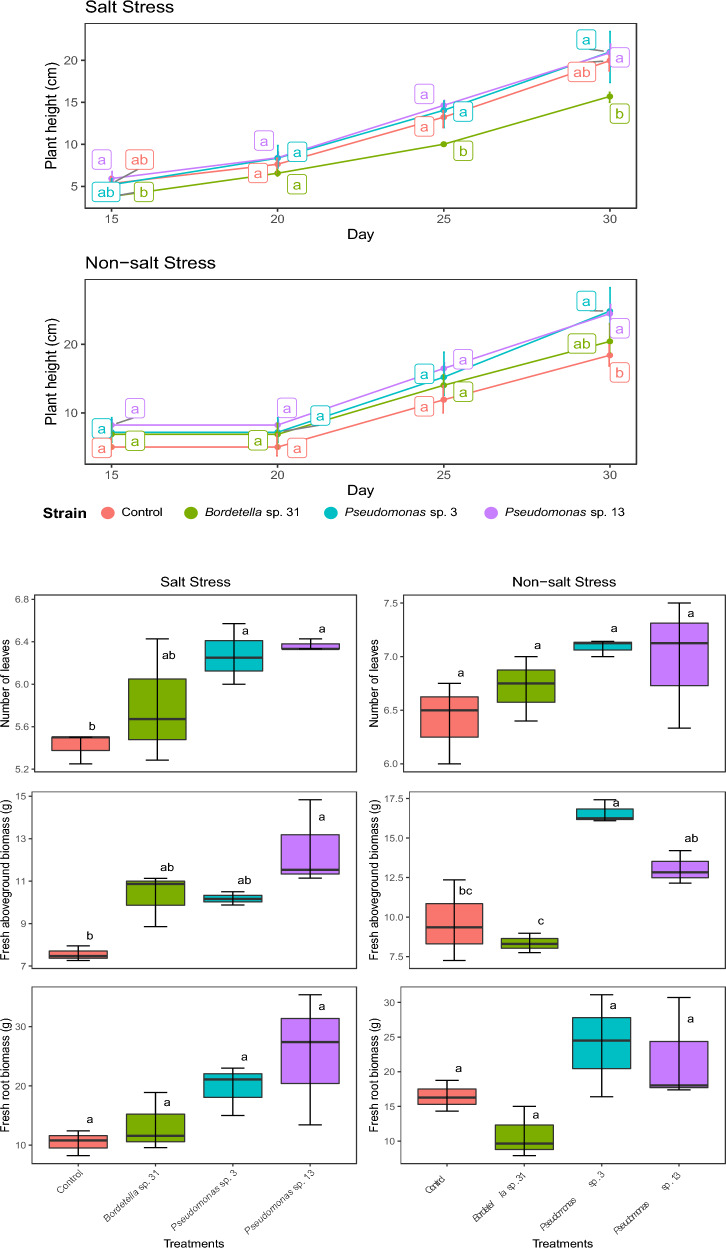
Plant height, number of leaves, fresh aboveground biomass (g), and fresh root biomass (g) in *R. sativus* seeds inoculated with the strains *Pseudomonas* sp. 03 (MW604823); strain *Pseudomonas* sp. 13 (MW604824); strain *Bordetella* sp. 31 (MW604826) and Control

We observed a distinct functional response on the morphological parameters of *R. sativus* inoculated with MW604823, MW604824 and MW604826, when planted under saline and non-salt stress (Table 4 and Fig. 3).

**Table 4:** Responses of morphological parameters of *R. sativus* inoculated with the strains MW604823, MW604824 e MW604826 grown in saline and non-saline soils

Isolate	Plant height (cm)	Leaf number	Aerial biomass (g)	Root number	Root biomass (g)
Saline soils					
Control	15.69 ± 1.13 a	5.42 ± 0.14 b	7.56 ± 0.35 b	4.83 ± 0.52 b	10.47 ± 1.25 c
MW604823	20.90 ± 0.90 a	6.27 ± 0.29 ab	10.18 ± 0.31 ab	6.77 ± 0.13 a	19.70 ± 1.62 b
MW604824	20.99 ± 3.21 a	6.37 ± 0.05 a	12.51 ± 2.03 a	6.78 ± 0.38 a	25.40 ± 0.69 a
MW604826	19.96 ± 1.06 a	5.76 ± 0.59 ab	10.29 ± 1.24 ab	6.10 ± 0.22 a	13.34 ± 1.57 c
Non-saline soils					
Control	20,42 ± 2,27 ab	6,42 ± 0,38 a	9,65 ± 2,56 bc	5,53 ± 0,32 b	16,45 ± 2,22
MW604823	24,46 ± 1,22 a	7,09 ± 0,08 a	16,59 ± 0,73 a	7,38 ± 0,44 a	24,00 ± 7,36
MW604824	24,83 ± 2,98 a	6,99 ± 0,60 a	13,06 ± 1,05 ab	7,08 ± 0,14 a	22,04 ± 7,52
MW604826	18,40 ± 1,54 b	6,72 ± 0,30 a	8,34 ± 0,62 c	7,03 ± 0,21 a	10,85 ± 3,69

Different letters mean tested treatments and Tukey’s post hoc tests with a confidence level of 5% (*p* < 0.05)

Our experiment showed that by inoculating MW604823, MW604824 and MW604826 in radish can promote plant gowth in saline soils, we can conclude that by observing a significant development in the number of leaves, roots and the total biomass weight (with a higher shoot and root volume). These results are presented in Table 4, and Fig. 3 and Table S4.

However, by analyzing the number of roots and biomass weight results of the inoculated plant in non-saline soils, we observed thatonly the strains MW604823 and MW604824, showed a greater response compared to the control. While strain MW604826 caused a unfavorable effect on plants in non-saline soil (Table S5).

**Fig. 4:**
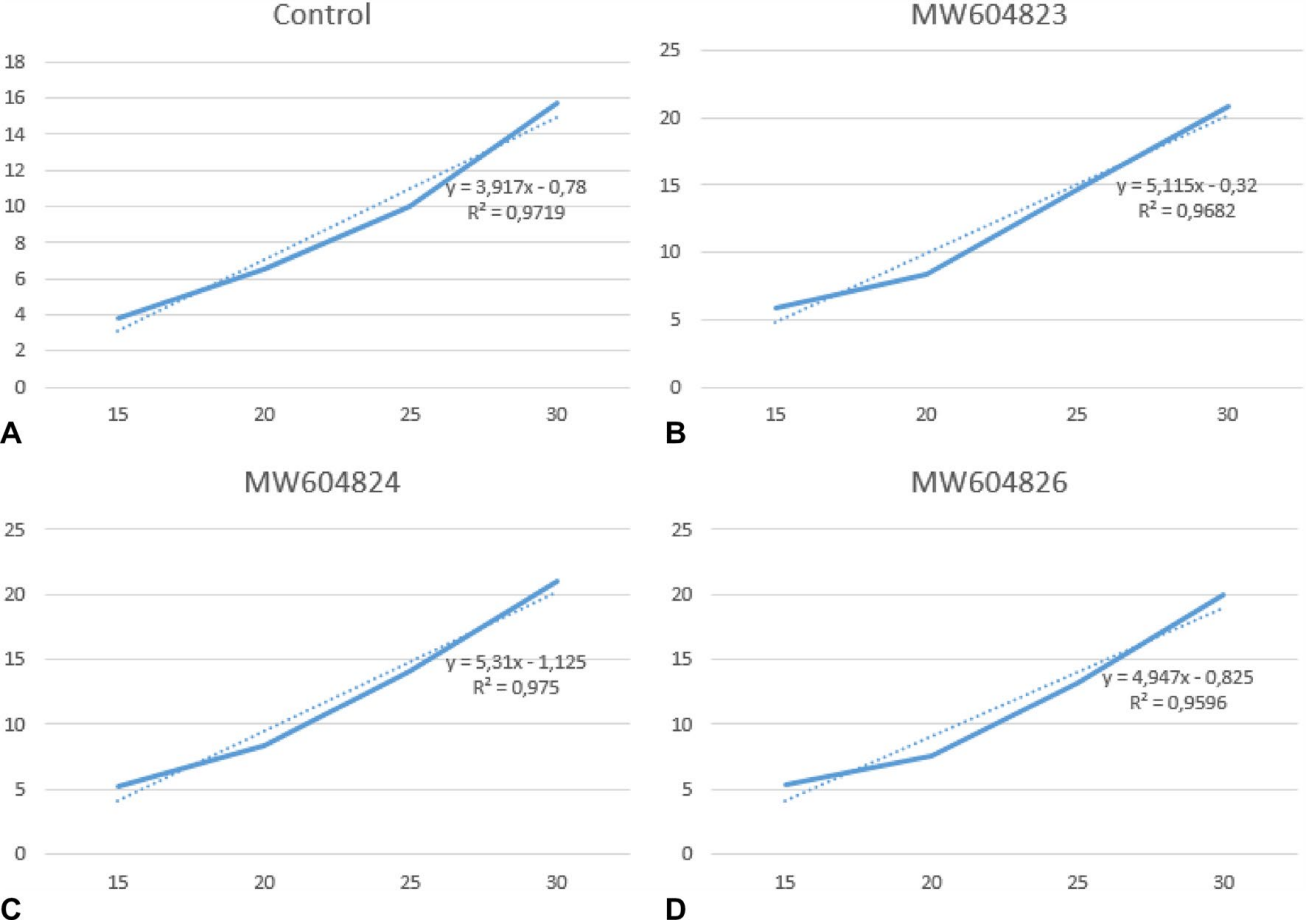
Linear regression and analysis of the growth curve of *R. sativus* plants throughout the 30 days of production in saline soils.** A**. Control;** B**. *R. sativus* inoculated with *Pseudomonas* sp. 03 (MW604823);** C**. *R. sativus* inoculated with *Pseudomonas* sp. 13 (MW604824);** D**. *R. sativus* inoculated with *Bordetella* sp. 31 (MW604826)

**Fig. 5:**
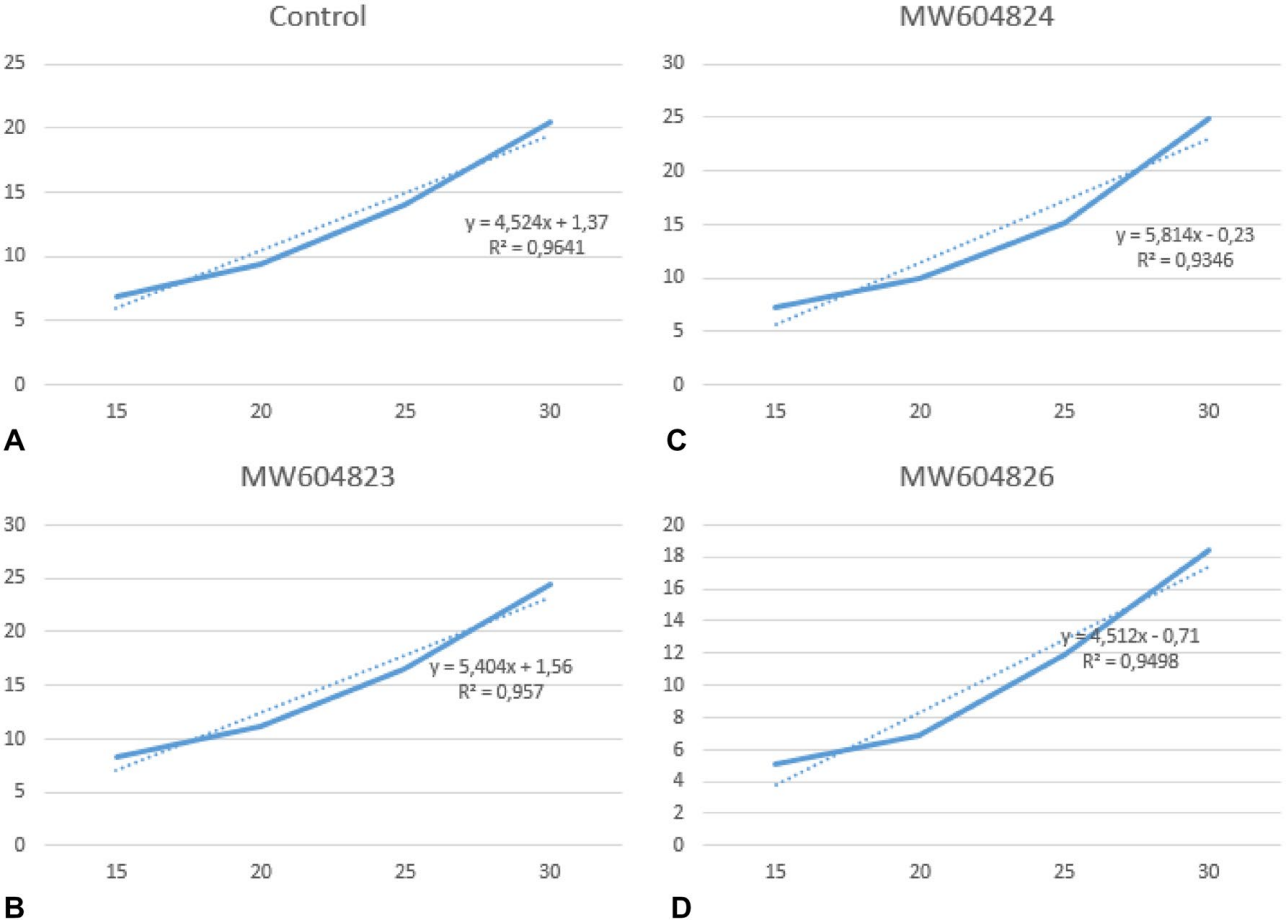
Linear regression and analysis of the growth curve of *R. sativus* plants throughout the 30 days of production in non-saline soils. **A.** Control; **B.** *R. sativus* inoculated with *Pseudomonas* sp. 03 (MW604823); **C.** *R. sativus* inoculated *Pseudomonas* sp. 13 (MW604824); **D**. *R. sativus* inoculated with *Bordetella* sp. 31 (MW604826)

The rate growth promotion (PGR) of the plant was considered individually for each treatment in both soils. Therefore, a linear regression analysis over time was performed to determine the degree of slope of the curves as displayed in Figs. 4 and 5.

In saline soils, the inoculation of MW604823, MW604824 and MW604826 promoted a growth rate of 30.69, 35.80 and 26.34%, respectively. Whereas in non-saline soils the growth rate promotion was lower, with a range of 19.46, 28.43 and 0.22%, respectively. By analyzing the difference between the conditions in which the microbial strains were submitted, we concluded that the three isolates benefit from the presence of salt to increase their functional performance. When observing the interaction of the isolates with the plants, it was made evident by obtaining a deleterious effect on plant growth by the strain identified as *Bordetella* sp.

## Discussion

The salt stress is an environmental problem that reduces the productivity of agricultural crops due to the effect caused by ionic and osmotic disturbances that plants undergo when exposed to these environments. The inoculation of soybean salt with *Bradyrhizobium japonicum* USDA 101 and *Pseudomonas putida* TSAU1 synergistically improved the plant’s tolerance through altering root system structure facilitating nitrogen, phosphorus acquisition, and nodule formation [39].

The use of PGPR in saline conditions is an biotechnologic alternative to improve the crop yields. For this reason, in this study, three of the bacteria that exhibited PGPR characteristics (produce IAA, solubilize phosphates and possess ACC deaminase activity) from salt-stressed regions were identified: The strain MW604823 (*Pseudomonas* sp.) that, according to its phylogenetic origin, shared 87% homology with *Pseudomonas hunanensis* LV^T^ (JX545210); the MW604824 strain (*Pseudomonas* sp.), that shared 96% phylogenetic origin with *Pseudomonas* LBME_s^T^ (LBME01000002) and the strain MW604826 (*Bordetella* sp.), that shared 41% of its phylogenetic origin with *Bordetella tumbae* T6713-1-3b^T^ (LC053656) and *Bordetella muralis* T6220-3-2b^T^ (LC053647) (Table 2). All of the three strains grew at different salt concentrations (5%, 7.5%, and 10% NaCl) (Fig. S1).

The three isolates belong to the phylum Proteobacterium which is predominant in the rhizosphere of *Prosopis limensis* [40]. In another study, *Pseudomonas plecoglossicida* RGK was isolated from the rhizospheric soil of the turmeric plant and was its capacity to solubilize phosphate, zinc and potassium was assessed, as well as its potential to produce indole acetic acid, siderophores, nitrogen fixation, ammonia, hydrogen cyanide (HCN) and exopolysaccharide synthesis [41].

Similar studies have been reported showing that PGPR with ACC deaminase activity had effects on early nodulation in *Medicago sativa* [42]. A research group in Indonesia performed an in vitro screening, where one isolate (designated as R2.1) was able to produce siderophores, IAA and ACC deaminase which by 16S rRNA sequencing was identified as closely related to *Bordetella muralis* [43]. The production of indoles mediated by bacteria isolated from plants and different environments promote plant growth by increasing IAA synthesis [44] IAA was synthesized at its highest levels by strain *Pseudomonas fluorescence* PGPR-7 with 123.1 μg mL^−1^ [45], which may be directly influence the growth of *R. sativus* inoculated with the bacteria in the present study, which explains the raised performance in seed germination rate, plant growth and development after the 30 days of production. The result obtained in the present work corroborates with previous reports in respect to the fact that. IAA promoted the growth of *R. sativus* when inoculated with plant growth promoting bacteria such as *Lactobacillus* sp. *And Pseudomonas putida*, that were subjected to different concentrations of NaCl and increased radicle length compared with non-inoculated seeds [46].

Our work reinforces the advantage inoculating PGPRs in plants of saline soils. Also, our group described the potential of three possible new species of the Proteobacterium phylum to solubilize and make available phosphorus, that is normally unavailable to the crop.

Our biochemical tests results prove the that employing the PGPR microorganisms (MW604823, MW604824, MW604826) as to promote the development of plants in saline soils is beneficial. This technology can be applied in the productive management of the culture of R. sativus (radish) as natural, economic and viable technique, and can be beneficial even for the production of sustainable agribusiness. In the light of our findings, more studies relied on the the inoculation of these strains in other agricultural crops should be carried on.

The germination is a natural process that occurs in the presence of phytohormones such as cytokinin, IAA and gibberillin, which represent the main hormones responsible for the germination process of plants [47]. *Pseudomonas* sp. strain MW604823, *Pseudomonas* sp. strain MW604824 and *Bordetella* sp. strain MW604826 showed efficiency in inducing germination of *R. sativus* seeds under saline stress conditions with with increased greater than 100% (Table 3). This was a consequence of the ability of the three species to produce IAA, that were verified in the biochemical tests (Table 2). Thus, the IAA produced by strains played an essential role in the cell elongation process and promoted plant growth and development.w

The results obtained corroborates with previous reports [20, 47, 48], that concluded that PGPR can produce a protective agent for the plant against environmental stress agents, especially saline, where the environment exerts the osmotic and water pressure from the plant. Probably, the production of phytohormones and exopolysaccharides was responsible for the increased germination rate of *R. sativus* in the present work.

The application of microbial biotechnology by the PGPR inoculation proved to be efficient in inducing *R. sativus* plants’ tolerance and growth in saline environments (Fig. 4), as well as promoting plant growth in non-saline soils (Fig. 5). Our results shows three bacterial isolates that are able to increase the growth promotion rates, number of leaves and roots, and total biomass (leaves and roots) of *R. sativus* plants (Table 4). Among the various mechanisms by which PGPRs can improve the tolerance of plants to salinity, their high antioxidant activity is very significant, as previously described for *Pseudomonas putida* strain NTM22 and *Pseudomonas cedrine* NTCC12, that are able to grow when exposed to concentrations of 2, 4 and 6% NaCl [49].

The literature confirms that the inoculation of *Pseudomonas* spp. in plants under salinity conditions reveal the mechanism of production of AIA and ACC deaminase, which act together forming a viable strategy to guarantee productivity, as they allow greater development in root volume [39, 50–52]. When comparing the growth of inoculated plants to the control in saline and non-saline soils, we can observe that the effective growth occurred mainly in saline soils, this performance may be linked to the need/dependence that these microorganisms have on the saline environment.

The increased salinity dependence was evident for the *Bordetella* sp. (MW604826) which had a lower performance than the control when subjected to a non-saline environment (Table 4 and Fig. 5).

A recent global pandemic and the resulting increase of food demand, as well as the decline of agricultural productivity, have led to a deficit in food production, and soil salinity has been a major part of the problem, promoting a decrease in crop yield and quality. The most environmentally sustainable methods are the most profitable, and the application of PGPR bacteria is a superior alternative for the plant growth and protection, as they accelerate the seed germination and improve growth. Furthermore, as demonstrated in the present study that PGPR bacteria are a source of hormones involved in the development of agricultural crops subjected to salt stress and can can influence and achieve better biochemical performance in plants.

## Conclusion

We conclude that among the 78 isolates from *P. limensis* rhizosphere and root, strains 03, 13 and 31 can be considered new species. Molecular characterization classified strains 03, 13 and 31 as two *Pseudomonas* sp. and a *Bordetella* sp. respectively. The biochemical performance and the inoculation that we applied of the three isolates in plants of *R. sativus*, prove the potential of using these strains as a source of products for the development of new biofertilizer compounds for saline environments.

## Supplementary Information

Below is the link to the electronic supplementary material.

Supplementary file1 (DOCX 3487 KB)

## Data Availability

All metadata, and material Supplementary will be made available for publication.
