# Supplementary material for: Rhizobacterial Isolates from Prosopis limensis Promote the Growth of Raphanus sativus L. Under Salt Stress
Source: Curr Microbiol. 2023 Jul 5;80(8):269. doi: 10.1007/s00284-023-03379-w (PMC10319673; doi:10.1007/s00284-023-03379-w)
Supplement: Supplementary file 1 — Supplementary file1 (DOCX 3487 KB) [file 284_2023_3379_MOESM1_ESM.docx]

**Rhizobacterial isolates from *Prosopis limensis* promote the growth of *Raphanus sativus* L. under salt stress**

**Current Microbiology**

Rene Flores Clavo ^a,b,c^*[](https://orcid.org/0000-0002-4448-5981), Esteban Valladolid-Suyón ^d^[](https://orcid.org/0000-0003-1931-2840), Karin Reinoza-Farroñan ^d^[](https://orcid.org/0000-0003-4723-8318), Cristian Asmat Ortega ^b^[](https://orcid.org/0000-0002-2607-8012), Pedro Henrique Riboldi Monteiro ^b,c^[](https://orcid.org/0000-0002-0096-047X), Gladys A. Apaza-Castillo ^a,e^[](https://orcid.org/0000-0002-2540-3135), Gabriel Zuñiga-Valdera ^d^[](https://orcid.org/0000-0002-0224-0329), Fabiana Fantinatti Garboggini ^c^[](https://orcid.org/0000-0002-0096-047X), Sebastian Iglesias-Osores ^d^[](https://orcid.org/0000-0002-4984-4656), Carmen Rosa Carreño-Farfán ^d^[](https://orcid.org/0000-0003-0238-2666)

^a^ *Cesar Vallejo University, Perú.*

^b^ *Department of Biotechnology, Center for Research and Innovation in Multidisciplinary Active Sciences (CIICAM), Pasaje Real Street N° 174, Chiclayo, Lambayeque, Perú*

^c^ *Division of Microbial Resources of Chemical, Biological and Agricultural Pluridisciplinary Research Center (CPQBA), University of Campinas (UNICAMP), Alexandre Cazellato N° 999, Campinas, Paulínia, São Paulo, Brazil*

^d^ *Microbial Biotechnology Research Laboratory, Department of Microbiology and Parasitology. Pedro Ruiz Gallo National University, Juan XXIII N° 391 Street, Chiclayo, Lambayeque, Peru*

^e^ *Department of Genetics, “Luiz de Queiroz” College of Agriculture, University of São Paulo (USP), Piracicaba, São Paulo, Brazil*

**SUPPLEMENTARY INFORMATION**

**Table S1.** 78 cultures isolated from *P. limensis* rhizospheric soils.

| Strain  Code | Gram negative bacilli colonies  (N°) | Strains de *Pseudomonas* spp and *Bordetella* spp.  N° |
| --- | --- | --- |
| C14 | 6 | 1 |
| C13 | 5 | 3 |
| C10 | 4 | 3 |
| C8 | 3 | 3 |
| C15 | 3 | 2 |
| A17 | 3 | 2 |
| A6 | 2 | 0 |
| B6 | 2 | 0 |
| A7 | 2 | 2 |
| B7 | 2 | 2 |
| C7 | 2 | 0 |
| A9 | 2 | 0 |
| B9 | 2 | 1 |
| C9 | 2 | 2 |
| A10 | 2 | 1 |
| B10 | 2 | 1 |
| B12 | 2 | 2 |
| A13 | 2 | 1 |
| B13 | 2 | 2 |
| B14 | 2 | 1 |
| A15 | 2 | 1 |
| B15 | 2 | 0 |
| A16 | 2 | 2 |
| B16 | 2 | 2 |
| A1 | 1 | 0 |
| B1 | 1 | 0 |
| B4 | 1 | 1 |
| C4 | 1 | 1 |
| B5 | 1 | 0 |
| A8 | 1 | 1 |
| B8 | 1 | 0 |
| A11 | 1 | 0 |
| B11 | 1 | 1 |
| C11 | 1 | 0 |
| A12 | 1 | 0 |
| C12 | 1 | 1 |
| A14 | 1 | 0 |
| C16 | 1 | 0 |
| B17 | 1 | 1 |
| C17 | 1 | 0 |
| B18 | 1 | 1 |
| C18 | 1 | 1 |
| Total 42 | 78 | 42 |

**Table S2**.  **Complete 16S rRNA sequences of valid names only EZBioCloud.**

| Strain | No. of bp | | BLASTn closest homolog  (accession #) organism | Completennes /Identity (%) | Variation radio |
| --- | --- | --- | --- | --- | --- |
| *Pseudomonas* sp. 03 | 1466 | [*Pseudomonas putida*](https://www.ezbiocloud.net/taxonomy?tn=Pseudomonas%20putida) NBRC 14164^T^ | | 100/(98.48) | 22/1450 |
| *Pseudomonas* sp. 13 | 1494 | | [*Pseudomonas monteilii*](https://www.ezbiocloud.net/taxonomy?tn=Pseudomonas%20monteilii)  NBRC 103158^T^  [*Pseudomonas plecoglossicida*](https://www.ezbiocloud.net/taxonomy?tn=Pseudomonas%20plecoglossicida) NBRC 103162^T^  [*Pseudomonas asiatica*](https://www.ezbiocloud.net/taxonomy?tn=Pseudomonas%20asiatica) RYU5^T^  [*Pseudomonas taiwanensis*](https://www.ezbiocloud.net/taxonomy?tn=Pseudomonas%20taiwanensis) BCRC 17751^T^  [*Pseudomonas entomophila*](https://www.ezbiocloud.net/taxonomy?tn=Pseudomonas%20entomophila) L48^T^ | 100/(98.97)  100/(98.97)  100/(98.97)  99.5/(98.96)  100/ (98.76) | 15/1450  15/1450  15/1450  15/1442    18/1450 |
| *Bordetella* sp. 31 |  | | [*Bordetella muralis*](https://www.ezbiocloud.net/taxonomy?tn=Bordetella%20muralis) T6220-3-2b^T^ | 100/ (97.76) | 32/1427 |

**Table S3. Complete 16S rRNA sequences of three strains.**

>*Pseudomonas* sp. 03 Accesion Number NCBI (MW604823)

TCAGATTGAACGCTGGGCGGCAGGCAGCTACACATGCAGTCGAGCGGATGACGGGAGCTTGCTCCTTGATTCAGCGGCGGACGGGTGAGTAATGCCTAGGAATCTGCCTGGTAGTGGGGGACAACGTTTCGAAAGGAACGCTAATACCGCATACGTCCTACGGGAGAAAGCAGGGGACCTTCGGGCCTTGCGCTATCAGATGTTTACGTCGGATTAGCTAGTTGGTGGGGTAATGGCTCACCAAGGCGACGATCCGTAACTGGTCTGAGAGGATGATCAGTCACACTGGAACTGAGACACGGTCCAGACTCCTACGGGAGGCAGCAGTGGGGAATATTGGACAATGGGCGAAAGCCTGATCCAGCCATGCCGCGTGTGTGAAGAAGGTCTTCGGATTGTAAAGCACTTTAAGTTGGGAGGAAGGGCATTAACCTAATACGTTAGTGTTTTGACGTTACCGACAGAATAAGCACCGACTTACTTGTGCCAGCAGCCGCGGTAATACAGAGGGTGCAAGCGTTAATCGGAATTACTGGGCGTAAAGCGGCGCGTAGGTGGTTTGTTAAGTTGGATGTGAAAGCCCCGGGCTCAACCTGGGAACTGCATCCAAAACTGGCAAGCTAGAGTACGGTAGAGGGTGGTGGAATTTCCTGTGTAGCGGTGAAATGCGTAGATATAGGAAGGAAACACCAGTGGCGAAAGGCGACCACCTGGACTGATACTGACACTGAGGTGCGAAAGCGTGGGGAGCAAACAGGATTAGATACCCTGGTAGTCCACGCCGTAAACGATGTCAACTAGCCGTTGGAATCCTTGAGATTTTAGTGGCGCAGCTAACGCATTAAGTTGACCGCCTGGGGAGTACGGCCGCAAGGTTAAAACTCAAATGAATTGACGGGGGCCCGCACAAGCGGTGGAGCATGTGGTTTAATTCGAAGCAACGCGAAGAACCTTACCAGGCCTTGACATGCAGAGAACTTTCCAGAGATGGATTGGTGCCTTCGGGAACTCTGACACAGGTGCTGCATGGCTGTCGTCAGCTCGTGTCGTGAGATGTTGGGTTAAGTCCCGTAACGAGCGCAACCCTTGTCCTTAGTTACCAGCACGTTATGGTGGGCACTCTAAGGAGACTGCCGGTGACAAACCGGAGGAAGGTGGGGATGACGTCAAGTCATCATGGCCCTTACGGCCTGGGCTACACACGTGCTACAATGGTCGGTACAGAGGGTTGCCAAGCCGCGAGGTGGAGCTAATCTCACAAAACCGATCGTAGTCCGGATCGCAGCTCTGCAAATCGACTGCGTGAAGTCGGAATCGCTAGTAATCGCG

AATCAGAATGTCGCGGTGAATACGTTCCCGGGCCCKACACCCGCCCGTCACACCATGGGAGTGGGTTGCACCAGAGTCCTAGTCTAACCTTCGGGAGGACGGTTACCACGGTGTGATTCATGACTGGGGTGAAGTC

> *Pseudomonas* sp. 13 Accession Number (MW604624)

GATTCCTGGCTCAGATTGAACGCTGGCGGCAGGCAGCTACCATGCAAGTCGAGCGGATGACGGGAGCTTGCTCCTTGATTCAGCGGCGGACGGGTGAGTAATGCCTAGGAATCTGCCTGGTAGTGGGGGACAACGTTTCGAAAGGAACGCTAATACCGCATACGTCCTACGGGAGAAAGCAGGGGACCTTCGGGCCTTGCGCTATCAGATGAGCCTAGGTCGGATTAGCTAGTTGGTGGGGTAATGGCTCACCAAGGCGACGATCCGTAACTGGTCTGAGAGGATGATCAGTCACACTGGAACTGAGACACGGTCCAGACTCCTACGGGAGGCAGCAGTGGGGAATATTGGACAATGGGCGAAAGCCTGATCCAGCCATGCCGCGTGTGTGAAGAAGGTCTTCGGATTGTAAAGCACTTTAAGTTGGGAGGAAGGGCAGTAAGTTAATACCTTGCTGTTTTGACGTTACCGACAGAATAGCACCGGCTAACTCTGTGCCAGCAGCCGCGGTAATACAGAGGGTGCAAGCGTTATCCGGGTACTGGGCGTAAAGCGCGCGTAGGTGGGTTTTGTTAAGTTGGATGTGAAAGCCCCCGGGCTCAACCTGGGAAATCTGCATCCAAAACTGGCAAAGCTAGAGTACGGTAGAGGGGTGGTTGGGAATTTCCTGTGTAGCGGTGAAATGCGTAGATATAGGAAGGAACACCCAGTGGCGAAGGGCGACCACCTGGACTGATACTGACACTGAGGTGCGAAAGCGTGGGGAGCAAACAGGATTAGATACCCTGGTAGTCCACGCCGTAAACGATGTCAACTAGCCGTTGGAATCCCTTGAGATTTTAGTGGCGCAGCTAACGCATTAAGTTGACCGCCTGGGGGAGTACGGCCGCAAGGTTAAAACTCAAATGAATTGACGGGGGCCCGCACAAGCGGTGGAGCATGTGGTTTAATTCGAAGCAACGCGAAGAACCTTACCAGGCCTTGACATGCAGAGAACTTTCCAGAGATGGATTGGTGCCTTCGGGAACTCTGACACAGGTGCTGCATGGCTGTCGTCAGCTCGTGTCGTGAGATGTTGGGTTAAGTCCCGTAACGAGCGCAACCCTTGTCCTTAGTTACCAGCACGTTATGGTGGGCACTCTAAGGAGACTGCCGGTGACAAACCGGAGGAAGGTGGGGATGACGTCAAGTCATCATGGCCCTTACGGCCTGGGCTACACACGTGCTACAATGGTCGGTACAGAGGGTTGCCAAGCCGCGAGGTGGAGCTAATCTCACAAAACCGATMAGTAGTCCGGATCGCWGTCTGCAACTCGAMTGCGTGAAGTCGGAATCGCTAGTAATCGCGAATCAGAATGTCGCGGTGAATACGTTCCCGGGCCTTGTACACACCGCCCGTCACACCATGGGAGTGGGTTGCACCAGAAGTMGCTAGTCTAACCTTCGGGAGGACGGTTACCAYGGTGTGATCATGACTGGGGTGAAGTCGACTAA

> *Bordetella* sp. 31 Accession Number (MW604826)

TTTACATGCAGTCGACGGCAGCGCGGACTTCGGTCTGGCGGCGAGTGGCGAACGGGGTAGTAATGGATCGGAACGTGCCTAGTAGCGGGGGATAACTACAATAAAGCGGAGCTAATACCGCATACGCCCTACGGGGGAAAGCGGGGGACCTTCGGGCCTCGCACTATTAGAGCGGCCGTATAAATTAGCTAGTTGGTGGGGTAAAGGCTCACCAAGGCGACGATCCGTAGCTGGTTTGAGAGGACGACCAGCCACACTGGGACTGAGACACGGCCCAGACTCCTACGGGAGGCAGCAGTGGGGAATTTTGGACAATGGGGGCAACCCTGATCCAGCCATCCCGCGTGTGCGATGAAGGCCTTCGGGTTGTAAAGCACTTTTGGCAGGAAAGAAACGGCCCTGGATAATATCTGGGGCAACTGACGGTACCTGCAGAATAAGCACCGGCTAACTACGTGCCAGCAGCCGCGGTATTACGTAGGGTGCAAGCGTTATTCGGAATTACTGGGCGTAAAAGCGTGCGCAGGCGGTTCCGGAAAGAAAGATGTGAAATCCCAGAGCTTAACTTTGGAAGCTGCATTTTTAACTACCGAGCTAGAGTGTGTCAGAGGGAGGTGGAATTACCGCGTGTAGCAGTGAAATGCGTAGAGATGCGGAGGAACACCGATGGCGAAAGCAGCCTCCTGGGATAACACTGACGCTCATGCACGAAAGCGTGGGGAGCAAACAGGATTAGATACCCTGGTAGTCCACGCCCTAAACGATGTCAACTAGCTGTTGGGGCCTTCGGGCCTTGGTAGCGCAGCTAACGCGTGAAGTTGACCGCCTGGGGAGTACGGTCGCAAGATTAAAACTCAAAGTAATTTGACGGGGCCCCGCACAAGCGGTGGATGATGTGATTTAATTCGATGCAACGCGAAAAACCCTTACCTACCCTTGAACATGTCTGGAATGCCGAAGAGATTTGGCAGTGCTCGCAAGAAAACTGGAACACAGGTCTTGCATGCCTGTTCGTCAGCTCGTGATCGTGAGATGTTGGGTTAAGTCCCGCAACGAGCGCAACCCTTGTCATTAGTTGCTACGAAAGGGCACTCTAATGAGACTGCCGGTGACAAACCGGAGGAAGGTGGGGATGACGTCAAGTCCTCATGGCCCTTATGGGTAGGGCTTCACACGTCATACAATGGTCGGGACAGAGGGTTGCCAAGCCGCGAGGTGGAGCTAATCCCAGAAACCCGATCGTAGTCCGGATCGCAGTCTGCAACTCGACTGCGTGAAGTCGGAATCGCTAGTAATCGCGGATCAGCATGTCGCGGTGAATACGTTCCCG

GGTCTTGTACACACCGCCCGTCACACCATGGGAGTGGGTTTTACCAGAAGTAGTTAGCCTAACCGCAAGGGGGGCGATTACCACGGTAGGATTCATGACTGGGGTGAAGTCGTAACAAG

**Table S4***.*  **Analysis of variance (ANOVA) for germination percentage of inoculated *R. sativus* seeds.** Data were calculated from three replicates twelve days after inoculation ***** shows statistical significance (p ≤ 0.05).

| **Source** | **Sum of squares** | **d.f** | **Mean square** | **F** | **Significance** |
| --- | --- | --- | --- | --- | --- |
| Between groups | 4695.83 | 3 | 1565.28 | 63.94 | * 0.00 |
| Within groups | 195.833 | 8 | 24.4792 |  |  |
| Total | 4891.67 | 11 |  |  |  |

**Table S5**.  **Analysis of variance (ANOVA) for Plant height, leaf number, aerial biomass, root number and root biomass in inoculated R. sativus seeds. *** shows statistical significance (p ≤ 0.05).

| **Source** | **Sum of squares** | **d.f** | **Mean square** | **F** | **Significance** |
| --- | --- | --- | --- | --- | --- |
| Plant height | 56.567 | 3 | 18.856 | 5.602 | * 0.023 |
| Leaf number | 1.784 | 3 | 0.595 | 5.185 | * 0.028 |
| Aerial biomass | 36.842 | 3 | 12.281 | 8.343 | * 0.008 |
| Root number | 7.542 | 3 | 2.514 | 20.89 | * 0.000 |
| Root biomass | 400.924 | 3 | 133.641 | 74.477 | * 0.000 |

| Soil Non-Salt stress | | | | | | | | | |
| --- | --- | --- | --- | --- | --- | --- | --- | --- | --- |
| Repetition | **Treatments** | **15 days height** | **20 days height** | **25 days height** | **30 days height** | **# leaves** | **weight aerial biomass** | **# roots** | **root weight** |
| I | *Pseudomonas* sp 3 | 8,99 | 12,90 | 17,30 | 25,80 | 7,13 | 16,25 | 7,00 | 16,40 |
| I | *Pseudomonas* sp 13 | 9,31 | 12,67 | 18,85 | 28,26 | 7,13 | 14,21 | 7,25 | 30,71 |
| I | *Bordetella* sp 31 | 6,90 | 9,07 | 13,98 | 19,93 | 6,75 | 8,30 | 7,25 | 7,92 |
| I | Testigo | 8,48 | 7,23 | 15,58 | 19,50 | 6,75 | 9,35 | 5,75 | 16,28 |
| II | *Pseudomonas* sp 3 | 8,23 | 10,56 | 16,39 | 24,19 | 7,14 | 17,43 | 7,29 | 24,50 |
| II | *Pseudomonas* sp 13 | 5,73 | 8,23 | 12,57 | 23,43 | 7,50 | 12,15 | 7,00 | 17,38 |
| II | *Bordetella* sp 31 | 4,43 | 6,18 | 11,78 | 18,43 | 7,00 | 7,75 | 6,83 | 9,64 |
| II | Testigo | 7,38 | 8,10 | 12,95 | 18,75 | 6,00 | 12,35 | 5,17 | 18,75 |
| III | *Pseudomonas* sp 3 | 7,50 | 9,86 | 15,76 | 23,40 | 7,00 | 16,10 | 7,86 | 31,10 |
| III | *Pseudomonas* sp 13 | 6,53 | 9,08 | 14,22 | 22,82 | 6,33 | 12,83 | 7,00 | 18,03 |
| III | *Bordetella* sp 31 | 3,76 | 5,52 | 10,02 | 16,84 | 6,40 | 8,98 | 7,00 | 15,00 |
| III | Testigo | 4,83 | 9,10 | 13,57 | 23,00 | 6,50 | 7,25 | 5,67 | 14,33 |
| Soil Salt stress | | | | | | | | | |
| I | *Pseudomonas* sp 3 | 6,76 | 9,07 | 13,86 | 21,93 | 6,57 | 10,50 | 6,86 | 21,10 |
| I | *Pseudomonas* sp 13 | 4,50 | 7,20 | 12,00 | 17,36 | 6,43 | 11,14 | 7,00 | 13,43 |
| I | *Bordetella* sp 31 | 4,03 | 6,60 | 12,00 | 20,57 | 5,67 | 10,87 | 6,29 | 11,57 |
| I | Testigo | 3,40 | 6,23 | 10,00 | 15,01 | 5,50 | 7,95 | 4,25 | 12,40 |
| II | *Pseudomonas* sp 3 | 5,43 | 7,95 | 14,83 | 20,45 | 6,00 | 10,17 | 6,83 | 15,00 |
| II | *Pseudomonas* sp 13 | 5,90 | 8,00 | 15,00 | 23,42 | 6,33 | 11,54 | 7,00 | 27,40 |
| II | *Bordetella* sp 31 | 5,81 | 8,34 | 14,00 | 18,74 | 5,29 | 8,86 | 6,14 | 9,57 |
| II | Testigo | 3,70 | 7,00 | 9,79 | 15,89 | 5,25 | 7,26 | 4,84 | 10,80 |
| III | *Pseudomonas* sp 3 | 5,59 | 8,19 | 15,24 | 20,31 | 6,25 | 9,88 | 6,63 | 23,00 |
| III | *Pseudomonas* sp 13 | 5,17 | 9,86 | 15,16 | 22,22 | 6,33 | 14,84 | 6,33 | 35,39 |
| III | *Bordetella* sp 31 | 6,17 | 7,96 | 13,71 | 20,57 | 6,43 | 11,13 | 5,86 | 18,88 |
| III | Testigo | 4,25 | 6,48 | 10,25 | 16,20 | 5,50 | 7,47 | 5,42 | 8,24 |

**Figures**

**
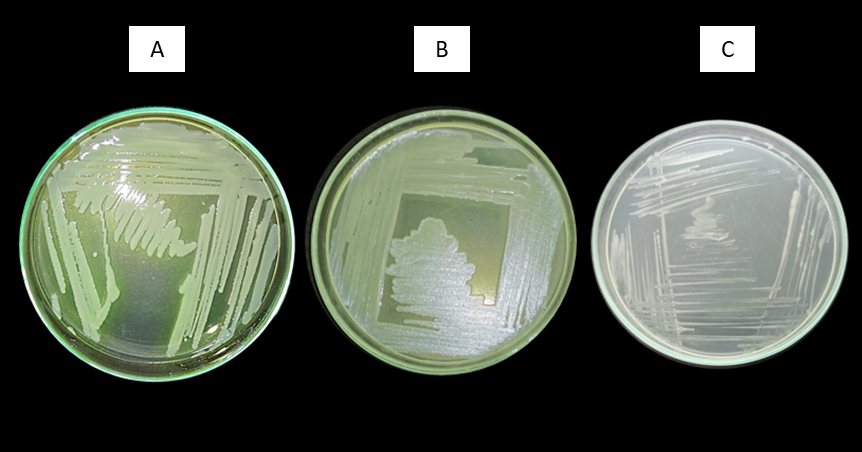
**

**Figure S1.** The phenotype of rhizobacterial isolates cultured on agar plates. From left to right A. Isolate 03 (MW604823). B. Isolate 13 (MW604824). C. Isolate 31 (MW604826).


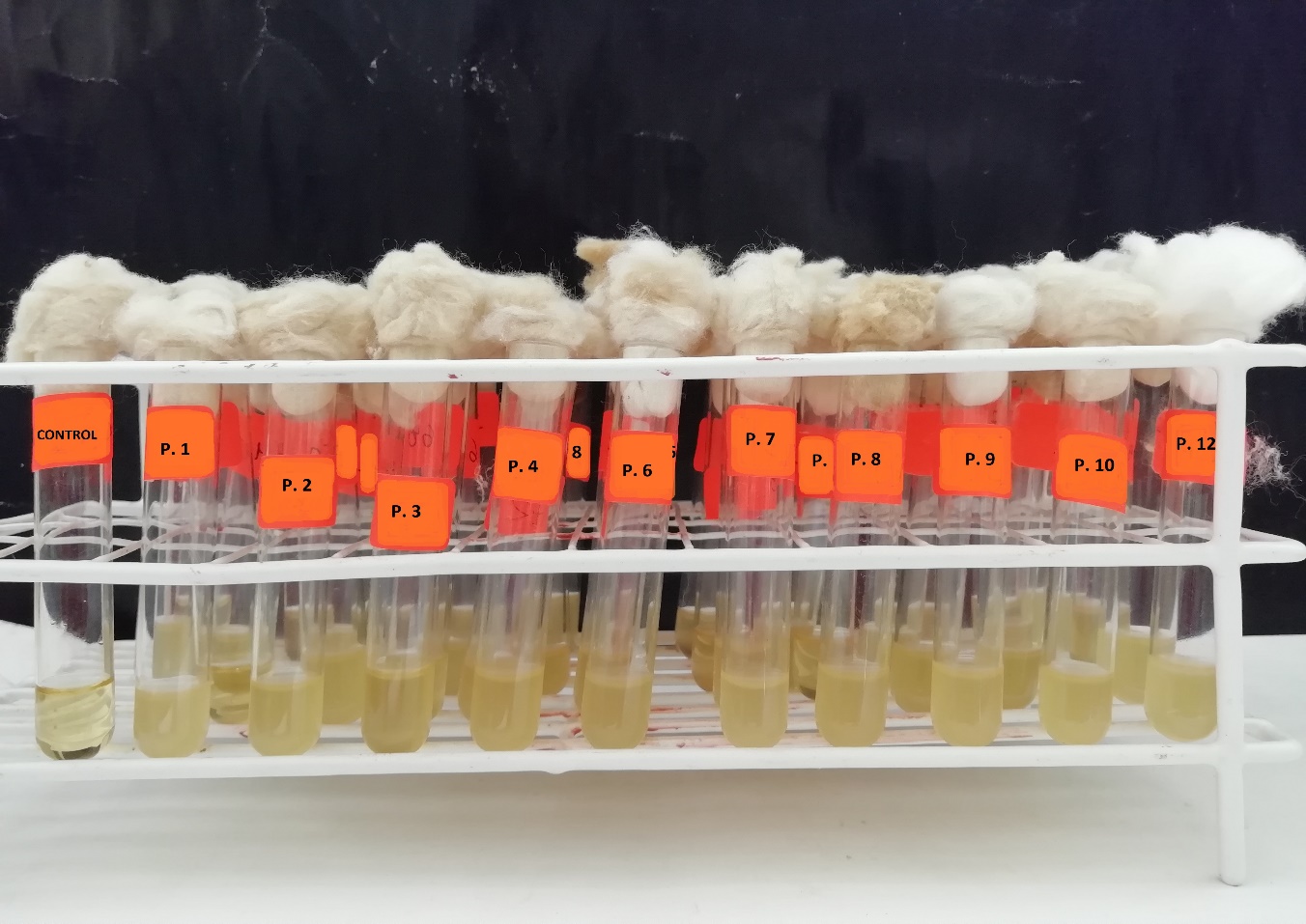


**Figure S2.** **Isolates’ tolerance to salt stress** All isolates 03, 13, and 31 were able to grow in Nutrient Broth media supplemented with 10 % of NaCl and showed differential turbidity in comparison with the control.


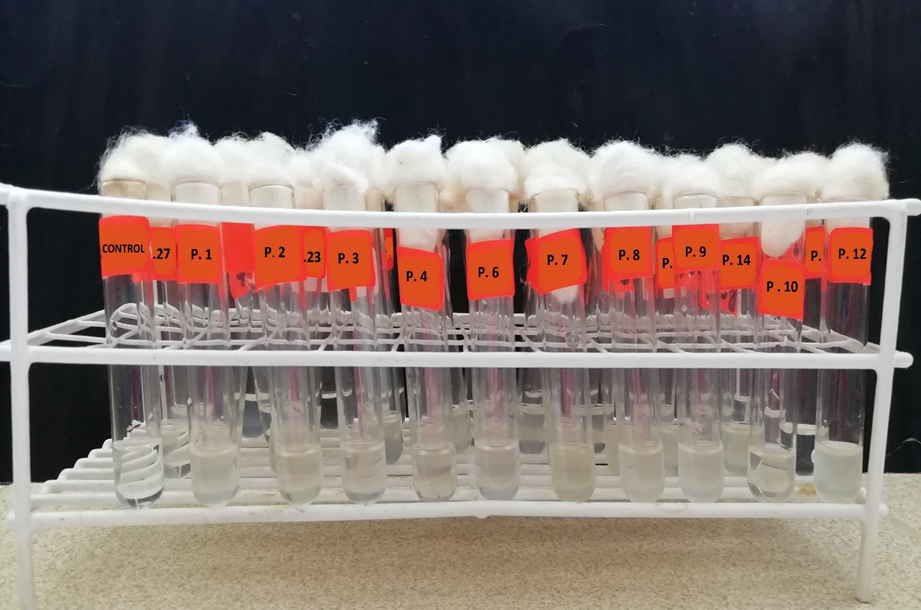


**Figure S3.** **ACC deaminase activity of rhizobacterial isolates.** All isolates 03, 13, and 31 were able to grow in DF media supplemented with 0.5 M of ACC and showed differential turbidity in comparison with the control.


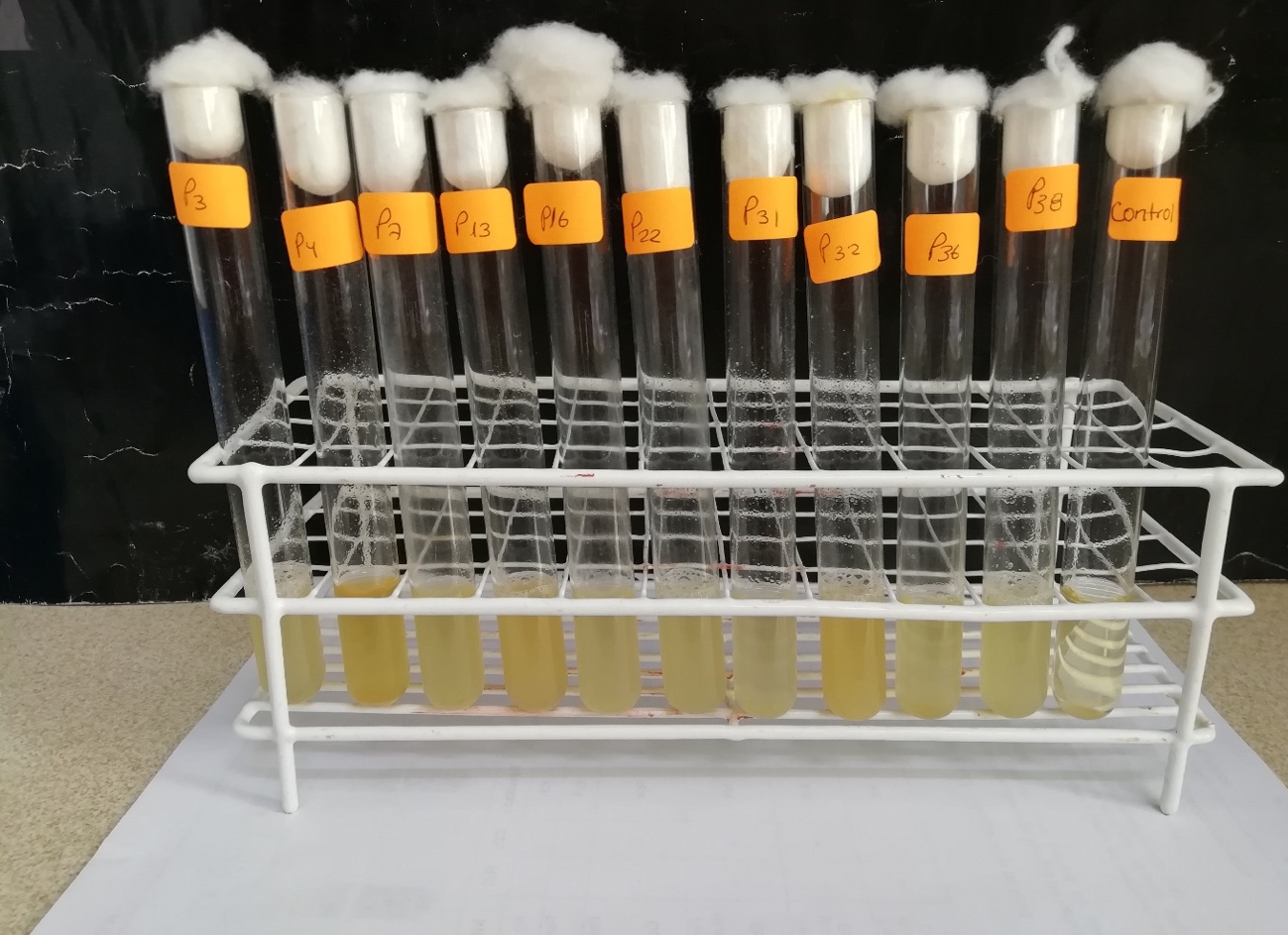


**Figure S4.** **Indole production of rhizobacterial isolates.** All isolates 03, 13, and 31 were able to grow in TSB media supplemented with 0.01 gL^-1^ of tryptophane and showed differential turbidity in comparison with the control.

***
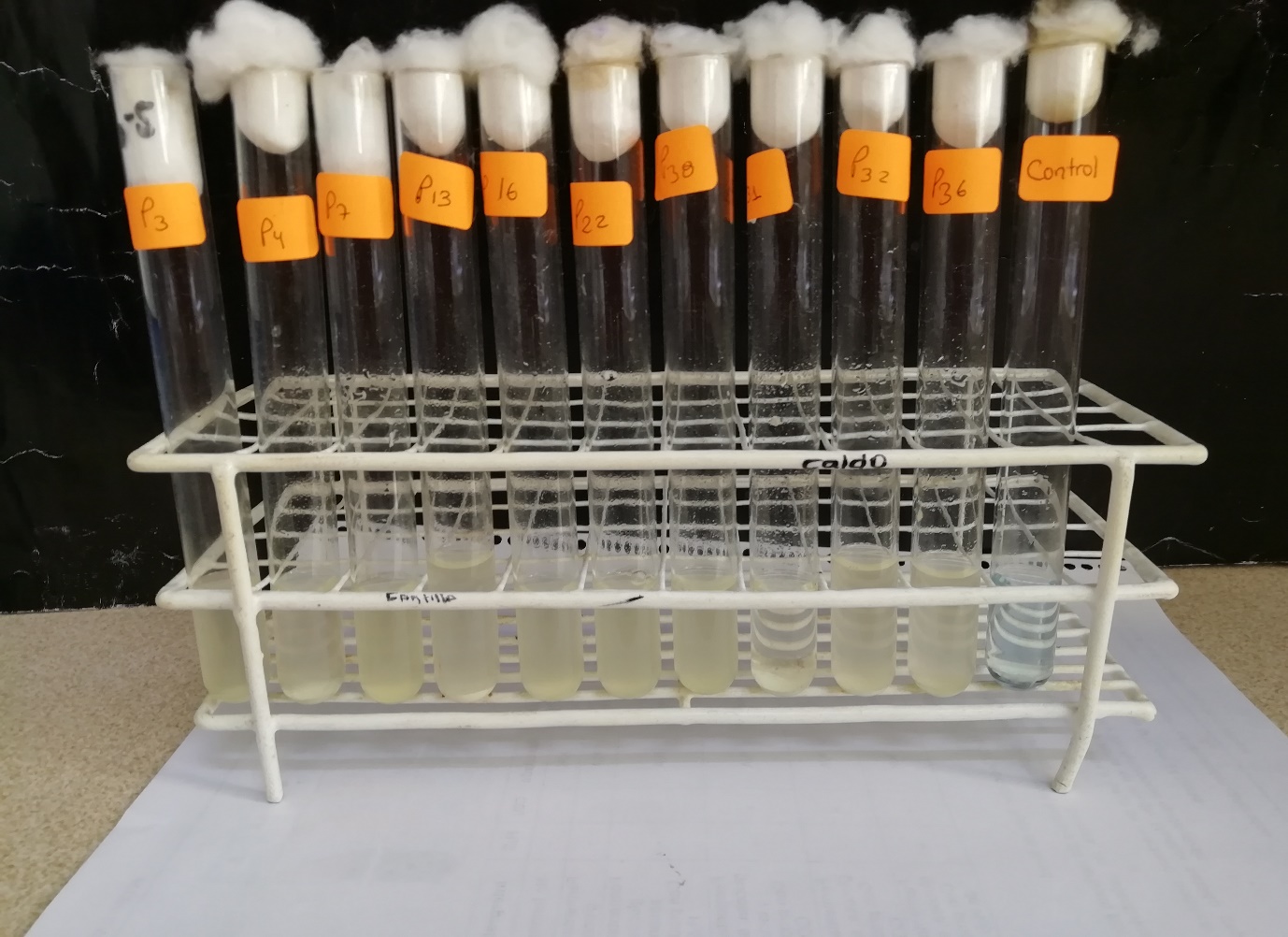
***

**Figure S5. Phosphate solubilization capacity** **of rhizobacterial isolates.** All isolates 03, 13, and 31 were able to grow in TSB media supplemented with 1 $\mathrm{gL}^{-1}$ of tricalcium phosphate and showed differential turbidity in comparison with the control.


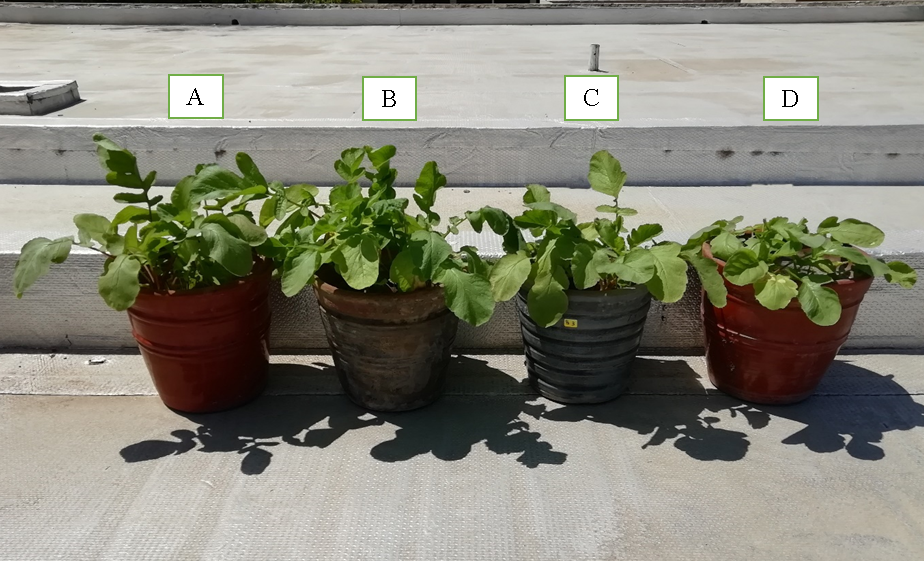


**Figure S6.** The phenotype of *R. sativus* plants cultivated in saline soils after thirty days of inoculation. From left to right A. Strain *Pseudomonas* sp. 03 (MW604823). B. Strain *Pseudomonas* sp. 13 (MW604824). C. Strain *Bordetella* sp. 31 (MW604826). D. Control.
